# Cardioprotective potential of mitochondria-targeted antioxidant, mito-TEMPO, in 5-fluorouracil-induced cardiotoxicity

**DOI:** 10.1007/s00280-023-04529-4

**Published:** 2023-03-30

**Authors:** Prasad Kisan Tambe, A. Jesil Mathew, Sanjay Bharati

**Affiliations:** 1 Department of Nuclear Medicine, Manipal College of Health Professions, Manipal Academy of Higher Education, Manipal, Karnataka India; 2 Department of Pharmaceutical Biotechnology, Manipal College of Pharmaceutical Sciences, Manipal Academy of Higher Education, Manipal, Karnataka India

**Keywords:** Cardiotoxicity, Chemotherapy, Mitochondrial oxidative stress, Mitochondrial dysfunction, Mitochondria-targeted antioxidant, Combinatorial therapy

## Abstract

**Purpose:**

The mitochondria-targeted antioxidants (MTAs) are known to offer protection against mitochondrial oxidative stress. The recent evidences support their role in mitigating oxidative stress-induced diseases, including cancer. Therefore, this study investigated cardioprotective potential of mito-TEMPO against 5-FU-induced cardiotoxicity.

**Methods:**

Mito-TEMPO was administered to male BALB/C mice (intraperitoneally, 0.1 mg/kg b.w. for 7 days) followed by intraperitoneal administration of 5- FU (12 mg/kg b.w. for 4 days). During this period, mito-TEMPO treatment was also continued. The cardioprotective potential of mito-TEMPO was assessed by evaluating cardiac injury markers, extent of non-viable myocardium and histopathological alterations. Mitochondrial functional status and mitochondrial oxidative stress were assessed in cardiac tissue. 8-OHdG expression and apoptotic cell death were assessed using immunohistochemical techniques.

**Results:**

The level of cardiac injury markers CK-MB and AST were significantly (*P* ≤ 0.05) decreased in mito-TEMPO pre-protected group which was further reflected in histopathology as decrease in the percentage of non-viable myocardial tissue, disorganization, and loss of myofibrils. Mito-TEMPO ameliorated mtROS, mtLPO and conserved mitochondrial membrane potential. Further, it had significantly (*P* ≤ 0.05) improved the activity of mitochondrial complexes and mitochondrial enzymes. A significant (*P* ≤ 0.05) increase in the level of mtGSH, activity of mitochondrial glutathione reductase, glutathione peroxidase, and mitochondrial superoxide dismutase was observed. A decreased expression of 8-OHdG and reduced apoptotic cell death were observed in mito-TEMPO pre-protected group.

**Conclusion:**

Mito-TEMPO effectively mitigated 5-FU-induced cardiotoxicity by modulating mitochondrial oxidative stress, hence may serve as a protective agent/adjuvant in 5-FU-based combinatorial chemotherapy.

## Introduction

5-FU is a key drug in several anticancer regimens [1]. Antitumor effects of 5-FU are primarily mediated by the suppression of thymidylate synthase (TS), which disrupts the intracellular deoxynucleotide pool essential for DNA replication. Subsequently, cytotoxic metabolites of 5-FU get incorporated into DNA which leads to its fragmentation and cell death [2].

Despite its prevalent use in chemotherapy, 5-FU monotherapies are reported to have significant toxic effects on heart [3]. The exact molecular mechanism of 5-FU-induced cardiotoxicity has not been fully elucidated so far however, role of oxidative stress-induced by reactive oxygen species and mitochondrial injury are implicated in multiple studies [1, 4]. 5-FU is reported to significantly hamper cardiac antioxidant defense system especially mitochondrial superoxide dismutase activity (MnSOD) and mitochondrial function [5, 6]. Studies also confirmed that 5-FU treatment to cardiomyocytes induces mitochondrial swelling, disorganization of cristae, vacuolization, and caspase-mediated cell death [7, 8].

Regardless the importance of oxidative stress in 5-FU-mediated toxicity, conventional antioxidants failed to mitigate toxic effects of anticancer agent [9]. Investigations have been carried out highlighting the use of conventional antioxidants to avoid side effects of this anticancer agent [4]. Although, conventional antioxidants proved helpful to certain extent but failed to show benefits in human clinical trials [10]. Surprisingly, in certain instances, conventional antioxidants increased the risk of lung cancer in smokers and worsened tumor burden in a mouse model of non-small cell lung cancer [11]. Several factors might have contributed towards the failure of conventional antioxidant therapy of which non-specific nature of these antioxidants and unavailability at the site of ROS production could be a major reason [12].

These results led us to hypothesize that targeting antioxidants to mitochondria may reduce the burden of cardiotoxic side effects of 5-FU chemotherapy. MTAs are the class of antioxidants which can target and accumulate within the mitochondria several folds to cytosol [13]. One of the MTAs, Mito-TEMPO, has demonstrated excellent mitochondrial superoxide and alkyl radical scavenging activities in several studies [14]. Its high antioxidant activity has dramatically reduced mitochondrial ROS production and oxidative stress in cardiovascular diseases, hepatic disorders, kidney diseases and neurodegenerative disorders [14]. Mitochondrial protection is proved successful in studies on other anticancer drugs such as doxorubicin where, administration of Mito-TEMPO to C57BL/6 mice protected them against doxorubicin-induced cardiotoxicity [15]. Therefore, considering possible involvement of mitochondria in cardiotoxicity generated by 5-FU, the present study intends to investigate cardioprotective potential of mito-TEMPO in 5-FU mediated cardiotoxicity.

## Materials and methods

### Chemicals and reagents

5-fluorouracil (5-FU) was procured from Sigma–Aldrich, USA. DCFH-DA, and Rhodamine 123. Rabbit 8-OHdG (bs-1278R) polyclonal antibody, Goat anti-rabbit IgG secondary antibody (65–6120), IL-6 rabbit polyclonal antibody (bs-0782R), IL-10 rabbit polyclonal antibody (PA5-85660) and TNF alpha rabbit polyclonal antibody (bs-2081R) were procured from Thermo Fisher Scientific (Rockford, USA). TUNEL assay kit (TACS-XL In Situ Apoptosis Detection Kit) was procured from R & D systems, USA. Other chemicals utilized in the current research were of the highest quality and obtained from local Indian firms.

### Animals and treatment protocol

Male BALB/c mice (25–30 g), 6–8 weeks old, were obtained from Institutional central animal facility. Animals were housed under controlled conditions (room temperature 25 ± 1 °C; humidity (65–80%); 12/12 h alternate light/dark cycle). All animals were fed normal pellet and water ad libitum. Prior to the start of the experiment, all animals were acclimatized for one week. All animal experiments were approved by Institutional animal ethics committee (IAEC/KMC/54/2021). Animal experimentation was conducted in compliance with CPCSEA (Committee for the Purpose of Control and Supervision of Experiments on Animals, Gov. of India) regulations, and institutional animal ethics committee guidelines.

**Fig. 1:**
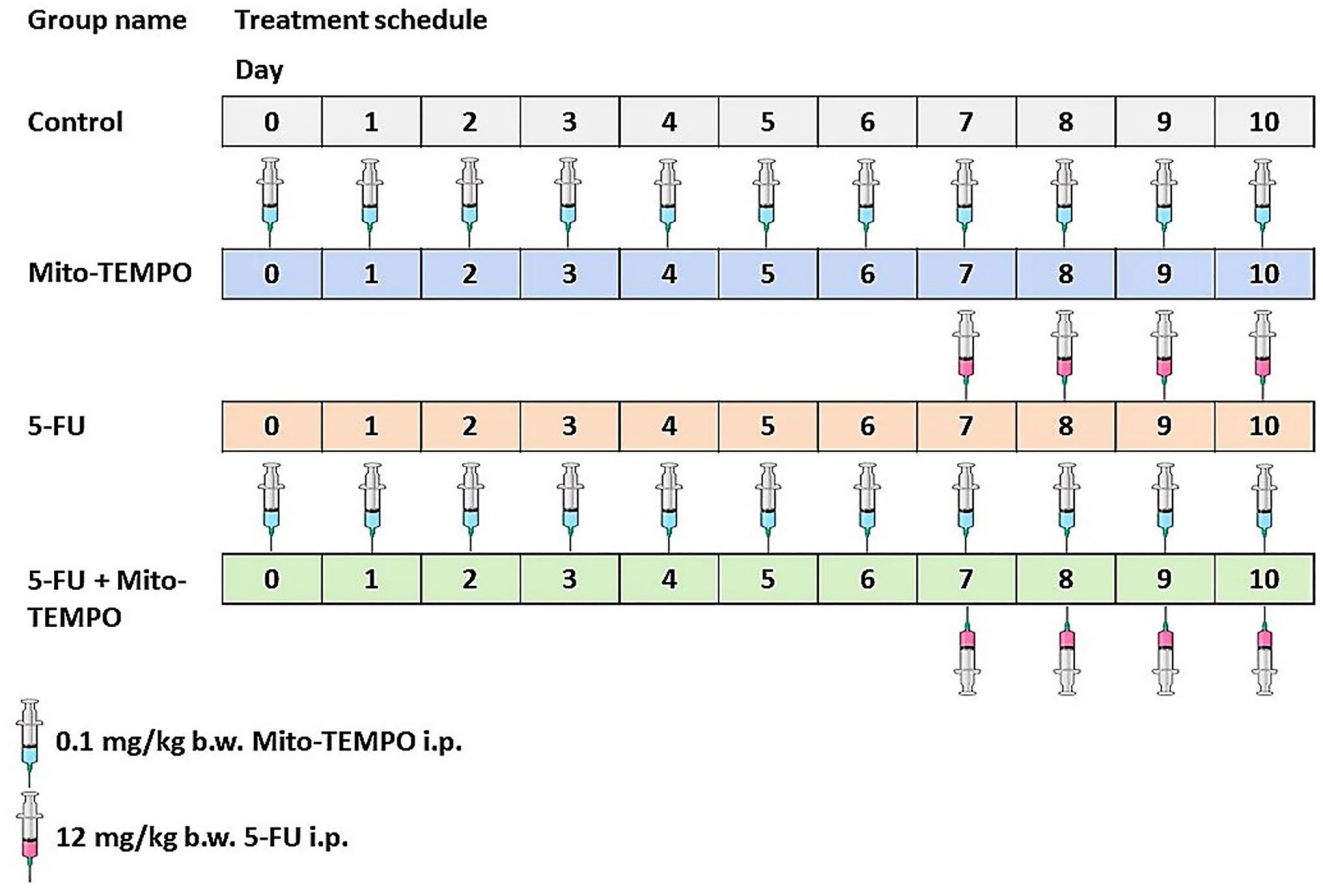
Schematic diagram for treatment protocol

Animals were randomly divided into four groups viz; Control, Mito-TEMPO, 5-FU, and 5-FU + Mito- TEMPO group. All animals were acclimatized for one week before starting the experimental protocol. 5-FU group received intraperitoneal injection (12 mg/kg b.w.) of 5-FU once a day for four consecutive days. Mito-TEMPO group received intraperitoneal dose of Mito-TEMPO (0.1 mg/kg b.w. dissolved in 0.9% NaCl (aq.)) daily till the termination of the study. 5-FU + Mito-TEMPO group animals received mito-TEMPO and 5-FU as described for 5-FU and Mito-TEMPO groups, respectively. Mito-TEMPO administration started one week prior to the 5-FU administration (Fig. 1). The animals were assessed for change in body weight, diet, and water intake daily. All mice were sacrificed after 24 h of the last injection of 5-FU and heart tissue samples were processed for further investigations.

### Cardiac injury marker

Blood samples of mice were obtained from retro-orbital plexus and the levels of serum CK-MB and AST were assessed using standard commercial kit (Liqui check, Agappe diagnostics, India) to confirm the induction of myocardial injury by 5-FU and the probable beneficial effect of mito-TEMPO treatment.

### Histopathological analysis

Histopathological changes in mice heart were determined by Hematoxylin and Eosin (H & E) staining. Briefly, tissue samples were fixed in formalin and processed for H & E staining as per the standard laboratory protocol [16]. The stained tissue sections were examined under light microscope (Lx 300, Labomed, USA).

### TTC staining for detection non-viable myocardial tissue

Non-viable myocardial tissue was assessed by 2,3,5-triphenyltetrazolium chloride (TTC) staining of heart tissue [16]. Briefly, excised heart tissues were washed with ice-cold 0.9% normal saline and thin transverse sections of the tissue were obtained. The tissue sections were incubated in TTC solution (10 mg TTC in 0.1 M sodium phosphate buffer) for 20 min. Further, tissue fixation was carried out by immersing the tissue in 10% formalin, overnight. The infarcted area was photographed and analyzed using Image J software (Version 1.53k, National Institute of Health, USA).

### 8-hydroxy-2-deoxyguanosine (8-OHdG) Immunohistochemistry

The immunohistochemistry for 8-OHdG was performed as described earlier [17]. Briefly, paraffin embedded cardiac tissue sections were deparaffinized and rehydrated in descending grades of alcohol. The epitope retrieval was carried out by immersing the slide in sodium citrate solution (10 Mm, pH 6.0). Slides were incubated in primary antibody 8-OHdG (diluted to 1:400) at room temperature. Further, tissue sections were incubated in HRP-labeled secondary antibody (diluted to 1:3000) for 1 h at room temperature and visualized under light microscope (Lx 300, Labomed, USA).

### TUNEL staining

For cell death analysis, TUNEL staining of cardiac tissue section was performed using commercially available kit as per manufacturer’s protocol. Briefly, deparaffinised and rehydrated tissue sections were covered with proteinase K and allowed to incubate at 37 °C for 15 min. After washing and quenching, slides were immersed in 1X TdT labeling buffer and covered with B-dNTP labeling reaction mixture at 37 °C for 30 min in a humidifying chamber. Further, slides were covered with Anti-BrdU antibody solution and incubated for 30 min at 37 °C. Further, slides were washed in PBS-Tween 20 solution for 2 min and immersed in Strep-HRP solution. Finally, after washing tissue section in PBS and deionized water slides were processed for counterstaining in methyl green and then observed under light microscope (Lx 300, Labomed, USA).

### Estimation of mitochondrial oxidative stress and activity of mitochondrial enzymes

Mitochondria from cardiomyocytes were isolated as described previously [18]. Mitochondrial oxidative stress was assessed in terms of mitochondrial lipid peroxidation (mtLPO) and mitochondrial reactive oxygen species (mtROS) as described earlier [17]. Briefly, levels of mtLPO were estimated by suspending mitochondrial fraction in Buege and Aust reagent, and centrifuged at 1000 × g (10 min). Optical density of the obtained supernatant was read at 532 nm and level of mtLPO was expressed as nmol MDA/mg protein (molar extinction coefficient 1.56 × 105 M^−1^ cm^−1^). The levels of mtROS were estimated by incubating the mitochondrial fraction in DCFH-DA (1.25 mM in methanol; 37 °C for 30 min). Optical density of the sample was read at excitation wavelength 500 nm and emission wavelength 520 nm in fluorimeter (FP8300, Jasco, USA). Further, activities of mitochondrial complex-I, complex-II, and complex-IV, malate dehydrogenase (MDH) and isocitrate dehydrogenase (IDH) in cardiac tissue were estimated according to the procedure described previously [19–21].

### Estimation of mitochondrial antioxidant defence status

Mitochondrial antioxidant defence status of mice heart was estimated in terms of activities of mitochondrial antioxidant defence enzymes. The ativity of mitochondrial reduced glutathione (mtGSH) was estimated by mixing 5,5′-dithiobis-2-nitrobenzoic acid (Ellman’s reagent) in 0.1 M phosphate buffer (pH 8) containing mitochondrial fraction [22]. Similarly, activity of mtGR was estimated by suspending mitochondrial fraction in substrate buffer (0.1 mM NADPH, 1 mM GSSG in 0.2 M phosphate buffer, pH 7.6), O.D. was noted at 340 nm for 3 min and activity of mtGR was expressed as nmol NADPH consumed/min/mg protein [23]. Further, activity of mtGPx was estimated by suspending mitochondrial fraction in reaction mixture (1 mM GSH, 0.22 mM NADPH, 5 mM EDTA, 50 mM Tris–HCl; pH 7.6). 0.22 mM tert-butyl hydroperoxide was added to the mixture, O.D. was noted at 340 nm for three minutes, and the activity of mtGPx was expressed as nmol NADPH oxidized/ min/mg protein [21]. The activity of MnSOD was estimated by suspending mitochondrial suspension in reaction mixture (0.2 mM pyrogallol, 1 mM EDTA, 50 mM Tris–HCl; pH 7.6) and O.D. of the sample was noted at 420 nm for three minutes. One unit enzyme activity was defined as the amount which decreased the autooxidation of pyrogallol by 50%. Enzyme activity was expressed as IU/min/mg protein [24].

### Estimation of mitochondrial membrane potential

Mitochondrial membrane potential in cardiac tissue was assessed according to the procedure described previously [19]. Briefly, mitochondrial fraction was suspended in the reaction mixture (pH 7.4) containing sucrose (150 mM), MgCl_2_ (4 mM), HEPES KOH (30 mM) and K_2_HPO_4_ (5 mM) and incubated at 37 °C for 5 min. Rhodamine 123 (5 μM) was added to initiate the reaction and the sample was read in fluorometer (FP8300, Jasco, USA) at wavelength of 507 nm (excitation) and 527 nm (emission).

### Inflammatory markers

Levels of inflammatory cytokines (IL-6, IL-10 and TNF-α) were assessed by ELISA technique. Briefly, antigen from cardiac tissues of mice was extracted as described earlier [25]. 96-well polystyrene flat-bottom ELISA plates (Himedia, India) were incubated with 50 μl of antigen overnight at 4 °C. After washing the plates with wash buffer (PBSTw), 1% bovine serum albumin was used as blocking reagent and100 μl of primary antibodies (diluted to 1:1000) of IL-6, IL-10 and TNF-α were added to respective well. Further, plates were incubated with horseradish peroxidase (HRP)-labeled goat anti-rabbit IgG secondary antibody (diluted to 1:3000) and O.D. was noted at 450 nm in ELISA plate reader.

### Protein estimation

Estimation of protein concentration present in mitochondrial fractions was carried out as described by previously [26]. The absorbance was noted at 620 nm using spectrophotometer.

## Statistical analysis

The Shapiro–Wilk and Levene’s test were used to determine the normality of the data and homogeneity of variance, respectively. The data were analyzed using one-way ANOVA followed by post hoc (Tukey’s HSD) for statistical significance. *P* ≤ 0.05 was considered statistically significant.

## Results

### Mito-TEMPO treatment protected against 5-FU-induced myocardial injury

**Fig. 2:**
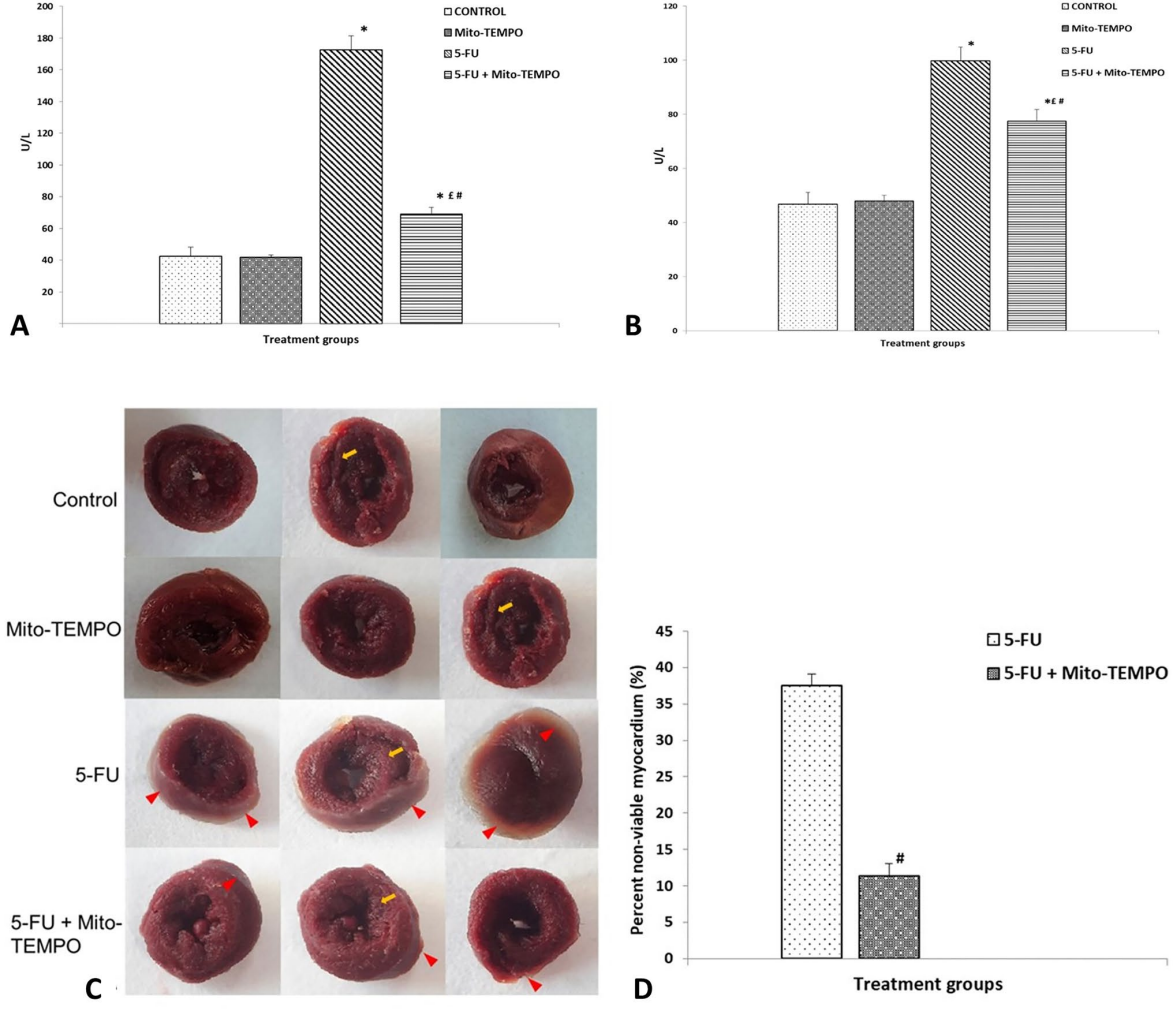
Effect of mito-TEMPO on 5-FU-induced cardiac injury. **A** The effect of Mito-TEMPO on the levels of serum CK-MB in various treatment groups. **B** The effect of Mito-TEMPO on the levels of serum AST in various treatment groups. **C** Cardioprotective action of Mito-TEMPO in terms of assessment of non-viable myocardial tissue in different treatment groups. Non-viable myocardial tissue was indicated by unstained, pale grayish zone (arrowhead). Interventricular septum (IS) was indicated by yellow arrow. **D** Graphical representation of percent non-viable myocardial tissue in different treatment group. (Data represented as mean ± SD. Data analysis was carried out using one-way ANOVA followed by post hoc test (Tukey’s HSD). *P ≤ 0.05 when compared to Control. ^£^*P* ≤ 0.05 when compared to mito-TEMPO. ^#^*P* ≤ 0.05 when compared to 5-FU)

Four-day exposure of 5-FU at a dose of 12 mg/kg b.w. to the mice did not show any significant changes in body weight, diet, and water intake of animals during the study period. However, it resulted in a marked alteration in the activities of serum cardiac injury markers; CK-MB and AST. The activities of both CK-MB and AST were significantly (*P* ≤ 0.05) elevated in 5-FU group as compared to Control group. The protective effect of mito-TEMPO in 5-FU + Mito-TEMPO group was evident as we observed significantly (*P* ≤ 0.05) lower values (2.52 and 1.28-fold) of serum activity of CK-MB and AST as compared to 5-FU group. However, protection was not enough to completely normalize the levels of CK-MB and AST to normal values when compared to Control and Mito-TEMPO group (Fig. 2A, B).

For assessing cardiac injury, we further performed TTC staining of heart sections obtained from different treatment groups to detect non-viable myocardial tissue. Morphology of 5-FU-treated heart showed pale, grayish unstained area indicative of non-viable myocardium (Fig. 2C). 5-FU challenged group showed 37.57% area of non-viable myocardium when compared to the Control group. However, pre-treatment with mito-TEMPO to 5-FU-treated animals significantly (*P* ≤ 0.05) reduced the area of non-viable myocardium to 11.37%. These results were indicative of cardioprotective action of mito-TEMPO (Fig. 2D).

**Fig. 3:**
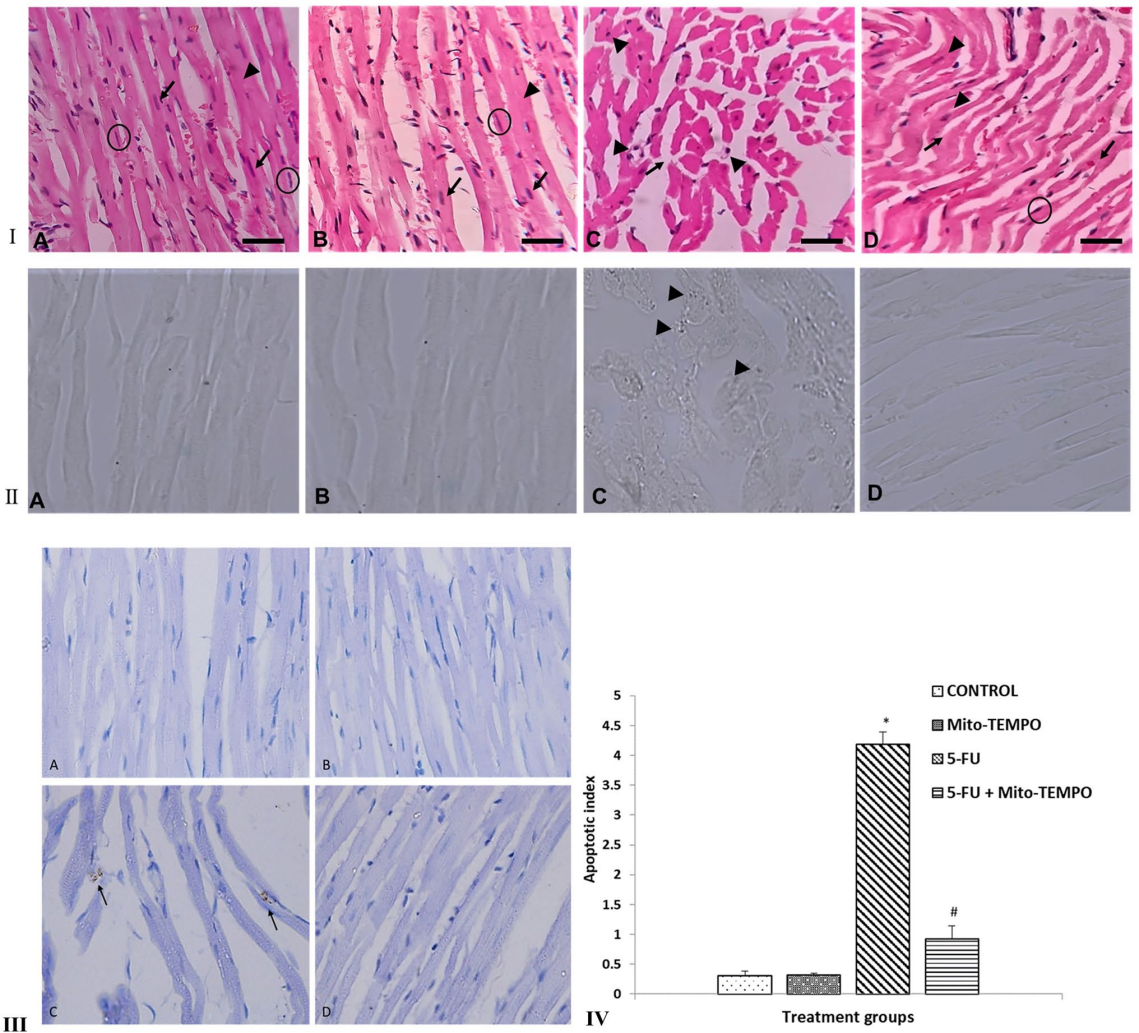
Effect of mito-TEMPO on histoarchitecture, oxidative DNA damage and apoptotic cell death in different treatment groups. **I** Photomicrographs of H and E-stained heart tissue of different treatment groups. **A** Control group showing normal heart muscles. **B** Mito-TEMPO group showing similar histoarchitecture as that of Control group. **C** 5-FU challenged group showing altered histoarchitecture. **D** Mito-TEMPO pre-treatment showing partially restored the cardiac histoarchitecture. **II** Immunohistochemical representation of effect of Mito-TEMPO on expression of 8-OHdG in various treatment groups. **A** Control group and **B** Mito-TEMPO group showing normal, light uniformly stained cardiomyocytes. **C** 5-FU group showing dark brown spots indicative of positive 8-OHdG immunostaining in cardiomyocytes (arrowhead). **D** Mito-TEMPO + 5-FU group showing normal uniformly stained cardiomyocytes. (Magnification 400×; Scale bar = 50 μm) **III** TUNEL staining of mice heart tissue in different treatment group. **A** Control group and **B** Mito-TEMPO group showing normal cardiac cells. **C** 5-FU group showing brown color TUNEL positive cells (black arrow). **D** Mito-TEMPO + 5-FU group showing normal cardiomyocytes **IV** Percentage of TUNEL positive cells in different treatment groups. (Data were presented mean ± SD. Data analysis was carried out using one-way ANOVA followed by post hoc test (Tukey’s HSD). *P ≤ 0.05 when compared to Control. # represents *P* ≤ 0.05 when compared to 5-FU)

The histopathological analysis further confirmed damage to cardiac tissue upon 5-FU treatment. H&E-stained heart tissue sections obtained from control group animals (Fig. 3IA) showed normal heart muscle fibers having striated branching and centrally placed nuclei (black arrow), acidophilic cytoplasm (arrowhead), and interstitial connective tissue with flat nuclei (circle). Mito-TEMPO group (Fig. 3IB) showed similar histoarchitecture when compared to Control group. 5-FU challenged animals showed disorganization, degeneration and loss of striations of myocardial fibers with clear spaces, indicative of intracellular edema (black arrow). The nuclei were pyknotic and present extracellularly (arrowhead) (Fig. 3IC). However, pre-treatment of mito-TEMPO effectively restored the histoarchitecture in terms of reappearance of straited branching of myocardial fibers, presence of single pale oval nucleus (arrowhead), interstitial connective tissue separated with flat nuclei (circle), decrease number of pyknotic nuclei and overall reduction in vacuolation (black arrow) (Fig. 3ID).

Further, DNA damage in 5-FU challenged animals was demonstrated by 8-OHdG immunostaining (Fig. 3II). The expression of 8-OHdG was increased in 5-FU group as compared to the Control group which might represent DNA damaging effect of 5-FU (Fig. 3IIC). Pre-treatment of mito-TEMPO to 5-FU challenged animals noticeably decreased the expression of 8-OHdG as compared to 5-FU group which indicated the protective action of mito-TEMPO against 5-FU mediated DNA damage (Fig. 3IID). Additionally, TUNEL stained sections of mice heart from different treatment groups were observed for cell death analysis (Fig. 3III). The overall percentage of TUNEL positive apoptotic cells (cells with brown nucleus) in 5-FU group was significantly increased (*P* ≤ 0.05) when compared to Control group. A significant reduction (*P* ≤ 0.05) in TUNEL positive cells was observed upon mito-TEMPO pre-treatment to 5-FU challenged animals (Fig. 3IV).

### Mito-TEMPO protected heart tissue from 5-FU-induced toxicity possibly by modulating the activities of ETC, TCA cycle enzymes and restoration of the mitochondrial membrane potential

A significant (*P* ≤ 0.05) reduction in the activity of mitochondrial respiratory complexes was observed in 5-FU treatment group when compared with Control group as shown in Table 1. Pre-treatment of mito-TEMPO to 5-FU challenged group significantly (*P* ≤ 0.05) enhanced the activity of mitochondrial complexes as compared to 5-FU group (Table 1). In addition, as compared to Control group, 5-FU administration dramatically decreased the activity of TCA cycle enzymes such as IDH and MDH. Mito-TEMPO protected group has shown significant (*P* ≤ 0.05) improvement in the activity of IDH and MDH as shown in (Table 1). The damage to heart mitochondria was further supported by significant (*P* ≤ 0.05) drop in the mitochondrial membrane potential (MMP) in 5-FU challenged group when compared with Control group. However, pre-treatment with mito-TEMPO significantly (*P* ≤ 0.05) raised the MMP when compared to 5-FU group (Table 1). Altogether, these findings highlighted the protective role of mito-TEMPO in mitigating the mitochondrial damage in 5-FU-induced cardiotoxicity.

**Table 1:** Effect of mito-TEMPO on 5-FU-induced mitochondrial dysfunction

Parameters	Control	Mito-TEMPO	5-FU	5-FU + Mito-TEMPO
Complex I (nmol NADH oxidized/min/mg mitochondrial protein)	27.42 ± 0.52	27.51 ± 0.80	16.66 ± 0.72^*^	21.98 ± 0.64^* £ #^
Complex II (nmol /min/mg mitochondrial protein)	115.97 ± 1.58	116.11 ± 1.52	69.07 ± 1.34^*^	89.38 ± 1.44^* £ #^
Complex IV (nmol NADH oxidized/min/mg mitochondrial protein)	0.414 ± 0.02	0.407 ± 0.01	0.191 ± 0.01^*^	0.299 ± 0.004^* £ #^
IDH (nmol NADH oxidized/min/mg mitochondrial protein)	52.90 ± 1.68	54.06 ± 1.24	36.95 ± 0.89^*^	48.59 ± 0.40^* £ #^
MDH (nmol NADH oxidized/min/mg mitochondrial protein)	191.60 ± 2.51	192.19 ± 4.45	87.38 ± 0.57^*^	124.88 ± 2.33^* £ #^
MMP (Relative intensity AFU)	103.98 ± 3.52	104.48 ± 3.28	55.42 ± 3.28^*^	88.04 ± 3.06^* £ #^
mtLPO (nmol /min/mg mitochondrial protein)	5.36 ± 0.17	5.23 ± 0.16	8.11 ± 0.15^*^	6.23 ± 0.18^* £ #^
mtROS (Relative intensity AFU)	55.22 ± 4.34	56.36 ± 2.95	98.04 ± 0.31^*^	75.26 ± 3.16^* £ #^

Data were presented as mean ± SD, analyzed by one-way ANOVA with subsequent application of post hoc test (Tukey’s HSD). *P ≤ 0.05 when compared to Control. ^£^*P* ≤ 0.05 when compared to mito-TEMPO. ^#^*P* ≤ 0.05 when compared to 5-FU

### Mito-TEMPO attenuated 5-FU-induced mitochondrial oxidative stress in mice heart

Since the damage to the respiratory mechanism and membrane potential of mitochondria could be associated with superoxide free radical formation, we were next encouraged to examine the effect of mito-TEMPO on mitochondrial oxidative stress. Expectedly, 5-FU-treated group showed significantly (*P* ≤ 0.05) increased levels mtLPO when compared to Control group. Pre-treatment with mito-TEMPO effectively reduced the levels mtLPO when compared to 5-FU group (Table 1).

Further, a significant (*P* ≤ 0.05) rise in the levels of mitochondrial reactive oxygen species (mtROS) was also observed in 5-FU challenged group when compared to control group. Mito-TEMPO pre-treatment to 5-FU challenged animals significantly (*P* ≤ 0.05) attenuated the levels of mtROS. Mito-TEMPO alone group had no change in the levels of mtROS when compared to Control group as demonstrated in Table 1. Collectively, these results underline the cardioprotective effect of mito-TEMPO by attenuating the mitochondrial oxidative stress in mice heart.

### Mito-TEMPO pre-treatment strengthened mitochondrial antioxidant defense system in 5-FU challenged animals

To emphasis on the role of O.S. in 5-FU-induced cardiotoxicity, we further checked the mitochondrial antioxidant defense status which helps in the maintenance of mitochondria redox homeostasis. Accordingly, to investigate the modulation occurring in the antioxidant defense enzymes, we estimated the activities of mitochondrial antioxidant enzymes. 5-FU challenged animals showed significant (*P* ≤ 0.05) decrease in mitochondrial GSH, GR, GPx and SOD when compared to Control group (Table 2). Mito-TEMPO + 5-FU group had shown significant (*P* ≤ 0.05) improvement in mitochondrial GSH, GR, GPx and SOD when compared to 5-FU group. These results strongly support the role of mito-TEMPO in conserving mitochondrial antioxidant defense system in 5-FU-induced cardiotoxicity.

**Table 2:** Effect of mito-TEMPO on mitochondrial antioxidant defense system

Parameters	Control	Mito-TEMPO	5-FU	5-FU + Mito-TEMPO
Mitochondrial GSH (nmol/mg mitochondrial protein)	5.39 ± 0.29	5.60 ± 0.11	2.37 ± 0.15^*^	3.99 ± 0.07^* £ #^
Mitochondrial GR (nmol/min/mg mitochondrial protein)	2.19 ± 0.07	2.24 ± 0.06	1.08 ± 0.02^*^	1.74 ± 0.03^* £ #^
Mitochondrial GPx (nmole/min/mg mitochondrial protein)	1.49 ± 0.04	1.50 ± 0.05	0.63 ± 0.05^*^	1.13 ± 0.05^* £ #^
Mitochondrial MnSOD (IU/min/mg mitochondrial protein)	15.53 ± 0.52	15.72 ± 0.74	8.72 ± 0.65^*^	12.44 ± 0.86^* £ #^

Data were presented as mean ± SD. Data analysis was carried out using one-way ANOVA with subsequent application of post hoc test (Tukey’s HSD). *P ≤ 0.05 when compared to Control. Ϯ represents *P* ≤ 0.05 when compared to Control. ^£^*P* ≤ 0.05 when compared to mito-TEMPO. ^#^*P* ≤ 0.05 when compared to 5-FU

### Modulatory effect of mito-TEMPO on inflammation in cardiac tissue

The concentrations of pro-inflammatory markers such as IL-6, IL-10 and TNF-α were analyzed by ELISA in cardiac tissues. As presented in Table 3, concentrations of IL-6, TNF- α were significantly (*P* ≤ 0.05) increased in 5-FU challenged animals when compared to control group. Conversely, significant (*P* ≤ 0.05) reduction in concentration of IL-10 in 5-FU challenged animals was observed when compared to control group. However, pre-treatment with mito-TEMPO significantly (*P* ≤ 0.05) normalized the levels of these cytokines in 5-FU-treated animals when compared to 5-FU group. The concentrations of inflammatory markers did not vary significantly in alone mito-TEMPO group as compared with Control. These results clearly demonstrated the anti-inflammatory role of mito-TEMPO in 5-FU-induced cardiotoxicity.

**Table 3:** Effect of mito-TEMPO on inflammatory markers in cardiac tissue

Parameters	Control	Mito-TEMPO	5-FU	5-FU + Mito-TEMPO
IL-6 proteins (OD/mg tissue protein)	4.40 ± 0.30	4.18 ± 0.12	6.61 ± 0.38 ^*^	5.60 ± 0.25^* £ #^
IL-10 proteins (OD/mg tissue protein)	4.39 ± 0.41	4.39 ± 0.30	2.79 ± 0.15 ^*^	4.19 ± 0.39 ^#^
TNF- α proteins (OD/mg tissue protein)	4.42 ± 0.49	4.25 ± 0.14	6.17 ± 0.41 ^*^	4.65 ± 0.88 ^#^

Data were presented as mean ± SD. Data analysis was carried out using one-way ANOVA with subsequent application of post hoc test (Tukey’s HSD). *P ≤ 0.05 when compared to Control. ^£^*P* ≤ 0.05 when compared to mito-TEMPO. ^#^*P* ≤ 0.05 when compared to 5-FU

## Discussion

The involvement of oxidative stress and reactive oxygen species (ROS) in 5-FU-related multiorgan toxicity has been highlighted earlier [27]. In recent years, the use of antioxidant treatment along with chemotherapy has remained a source of great debate. Regardless the importance of oxidative stress in chemotherapy-induced toxicity, the conventional antioxidants failed in mitigating the toxic effects of anticancer agents in clinical trials [10]. There are several factors responsible for this type of effect amongst which unavailability of antioxidant at the site of ROS production is considered main factor [28].

5-FU is an antimetabolite of nucleobase uracil which metabolizes in two ways i.e., anabolism and catabolism [29]. In anabolic pathway it primarily inhibits thymidylate synthase enzyme as its principal mechanism of action and creates thymine deficiency. Further, it gets converted into cytotoxic metabolite, fluorodeoxyuridine monophosphate (F-dUMP). This metabolite interferes the synthesis of DNA which leads to cancer cell death. The catabolic metabolism of 5-FU is generally linked to its toxic effects wherein, 5-FU is metabolized to fluor-citrate (FC) by the action of enzyme dihydropyridine dehydrogenase (DPD) that inhibits citrate metabolism and block the TCA cycle within mitochondria. This ultimately decreases the ATP production and alters mitochondrial membrane permeability, which leads to mitochondrial dysfunction and eventually cell death [29]. Further, 5-FU is also known to affect iron homeostasis, where 5-FU administration significantly raises Fe^2+^ levels in vivo [30]. The excessive iron accumulation in cardiomyocytes generates ROS via Fenton reaction, eventually leading to ferroptosis [30].

Mitochondria-targeted nitroxides, XJB-5-131 and JP-039 were shown effective in preventive ferroptotic cell death in HT-1080 fibrosarcoma cells, BJeLR cells and panc-1 cells [31]. Further, Suleiman et al., also demonstrated the anti-ferroptotic potential of XJB-5-131 against RSL3-induced ferroptosis in H9c2 cardiomyocytes [32]. Our present study aimed to investigate the possible role of mito-TEMPO, a mitochondria-targeted antioxidant, in preventing 5-FU-induced cardiotoxicity. Mito-TEMPO is a TPP + conjugated piperidine nitroxide. TPP + helps in mitochondrial accumulation and piperidine nitroxide mimics superoxide dismutase activity hence causing dismutation of mitochondrial superoxide free radical to hydrogen peroxide where mito-TEMPO is regenerated [14]. In the present study, administration of 5-FU to the animals resulted in a substantial increase in the cardiac injury markers CK-MB and AST which might indicated 5-FU-mediated cardiac toxicity. The direct relationship between these cardiac injury markers and severity of myocardial infarction is well-documented in the literature [33]. Furthermore, TTC staining which is a well-accepted approach for determining the extent of a non-viable myocardial tissue also supported the above findings [34]. The significant decrease in the levels of cardiac injury markers and percentage of non-viable myocardium in mito-TEMPO pre-treated group might indicated reduction in the cardiac toxicity by 5-FU. These results were in corroboration with findings of Ji et al., where MTA was shown successful in attenuating the levels of cardiac injury markers and myocardial infarction in myocardial ischemia–reperfusion injury [35].

Mitochondrial oxidative stress is thought to play a significant role in 5-FU-mediated cardiotoxicity. In an in-vitro study, an excessive production of mtROS is witnessed using fluorogenic Mito Probe after incubation of H9c2 cardiomyoblasts with 5-FU [5]. Additionally, 5-FU administration is demonstrated to decrease ATP production in cardiac mitochondria. This decrease ATP production is linked to the impaired oxidative phosphorylation in mitochondrial inner membrane after 5-FU treatment [36]. Literature evidence also highlights the role of 5-FU in inducing excessive mitochondrial autophagy which is confirmed by assessing expression of autophagy related proteins in cardiac tissues of rat [36]. The administration of 5-FU in this study also demonstrated a significant (*P* ≤ 0.05) increase in mtROS, mtLPO, dysfunction of mitochondrial complexes and decrease in mitochondrial membrane potential. These alterations might indicate towards the toxic effect of 5-FU in cardiac mitochondria. Mito-TEMPO co-treatment reduced mtROS and mtLPO that might represent its antioxidant potential. Furthermore, it improved the status of mitochondrial complexes and mitochondrial membrane potential.

Mitochondria have intrinsic antioxidant defence mechanism to protect itself from the oxidative stress produced during the metabolic activity [37]. However, chemotherapy-induced depression in the enzymatic activity of mitochondrial antioxidant enzymes such as mitochondrial SOD, GPx, GR and antioxidant GSH were demonstrated earlier [38]. Our results were in agreement with the above reports, wherein, 5-FU-challenged animals showed decreased activities of mitochondrial antioxidant enzymes such as GSH, GPx, GR and SOD. This might further lead to an increase in the mitochondrial ROS. However, pre-treatment with mito-TEMPO improved mitochondrial antioxidant defence. The effect of mito-TEMPO in mitigating mitochondrial oxidative stress was also confirmed from immunohistochemistry for 8-hydroxyguanosine (8-OHdG). 8-OHdG is a well-known oxidative DNA damage marker [39]. The continuous production of ROS and deficient antioxidant machinery in 5-FU-treated animals might lead to oxidative DNA damage which results into formation of adducts like 8-OHdG. The comparatively lower expression of 8-OHdG in mito-TEMPO co-treated group clearly indicated that it had provided enough antioxidant protection to the mitochondria of affected cardiac tissue.

Several chemotherapy-induced cardiotoxicity studies reported the important link between oxidative stress and inflammation. It is well reported in the literature that, following enhanced oxidative stress, involvement of the NF-κB transcription factor plays a pivotal role in triggering the production of cytokines [38]. IL-6 and TNF-α are important predictors of cardiovascular morbidity and death [40]. Our findings demonstrated a significant (*P* ≤ 0.05) increase in the concentrations of IL-6 and TNF-α in 5-FU-treated mice heart consistent with literature findings. Furthermore, our findings demonstrated that overexpression of TNF-α and IL-6 was also associated with a considerable decrease in IL-10 expression, one of the most essential anti-inflammatory immune regulatory cytokines [41].

The increased ROS in cardiomycytes predispose to ROS-mediated cell apoptosis by impeding membranal integrity which leads to mitochondrial damage. ROS-mediated outer mitochondrial membrane permeabilization and cytochrome c (an apoptosis-inducing agent) translocation, to the cytosol begin the intrinsic mitochondrial apoptotic pathway, where they activate caspase-dependent cell apoptosis [42]. TNF-α plays the most important role in initiation of apoptosis of cardiac cells mediated by TNF receptors TNFR1 and TNFR2. The activation of these receptors lead to the activation of caspase-8 which can further activate caspase-3, leading to apoptosis [43]. In our study, to assess the 5-FU-induced cardiac cell death we performed TUNEL assay. TUNEL assay results revealed a substantial increase in TUNEL positive cells in 5-FU-treated animals, indicating apoptotic cell death. These results were in agreement to those of Yuan et al. where TUNEL staining showed significant (*P* ≤ 0.05) increase in chemotherapy-induced cardiac cell apoptosis [34]. However, significant (*P* ≤ 0.05) decrease in apoptotic cardiomyocytes was observed when mito-TEMPO pre-treatment was given. Although, a significant cardio-protection is offered by mito-TEMPO against 5-FU-induced cardiotoxicity however, there is a scope for the further improvement in the extent of cardio-protection. The protection provided is not complete which could be due to several reasons. 5-FU-induced cardiotoxicity involves multiple mechanisms other than mitochondrial injury such as coronary vasospasm by decreased nitric oxide (NO) levels, direct myocardial toxicity by 5-FU metabolites like fluoroacetate and fluorocitrate which inhibits the citric acid cycle etc. [44]. Therefore, it may be possible to develop more safer chemotherapy regimen by simultaneously targeting other cardiotoxicity mechanism along with protecting mitochondria.

## Conclusion

In conclusion, our findings indicate that mito-TEMPO can effectively mitigate the 5-FU-induced cardiotoxicity by modulating mitochondrial oxidative stress. As per best of our knowledge this is the first report demonstrating protective role of mitochondria-targeted antioxidant in 5-FU-cardiotoxicity. Therefore, the encouraging results of this study may pave way for further investigations involving other chemotherapy drugs and different MTAs.

## Data Availability

Data are available and can be provided upon a request to corresponding author.

## Abbreviations

5-FU: 5-Fluorouracil
MTA: Mitochondria-targeted antioxidant
Mito-TEMPO: (2-(2,2,6,6-Tetramethylpiperidin-1-oxyl-4-ylamino)-2-oxoethyl) triphenylphosphonium chloride
8-OHdG: 8-Hydroxyguanosine
CK-MB: Creatine kinase-MB
AST: Aspartate aminotransferase
ROS: Reactive oxygen species
IL: Interleukin
TNF: Tumor necrosis factor
DCFH-DA: 2′-7′Dichlorofluorescin diacetate
TUNEL: Terminal deoxynucleotidyl transferase (TdT) dUTP Nick-End Labeling
TTC: 2,3,5-Triphenyltetrazolium chloride
NADH: Nicotinamide adenine dinucleotide
NADPH: Nicotinamide adenine dinucleotide phosphate
